# CancerMHL: the database of integrating key DNA methylation, histone modifications and lncRNAs in cancer

**DOI:** 10.1093/database/baae029

**Published:** 2024-04-12

**Authors:** Pengyu Du, Yingli Chen, Qianzhong Li, Zhimin Gai, Hui Bai, Luqiang Zhang, Yuxian Liu, Yanni Cao, Yuanyuan Zhai, Wen Jin

**Affiliations:** Laboratory of Theoretical Biophysics, School of Physical Science and Technology, Inner Mongolia University, 235 West Daxue Road, Hohhot 010021, China; Laboratory of Theoretical Biophysics, School of Physical Science and Technology, Inner Mongolia University, 235 West Daxue Road, Hohhot 010021, China; The State Key Laboratory of Reproductive Regulation and Breeding of Grassland Livestock, Inner Mongolia University, 235 West Daxue Road, Hohhot 010021, China; Laboratory of Theoretical Biophysics, School of Physical Science and Technology, Inner Mongolia University, 235 West Daxue Road, Hohhot 010021, China; The State Key Laboratory of Reproductive Regulation and Breeding of Grassland Livestock, Inner Mongolia University, 235 West Daxue Road, Hohhot 010021, China; Laboratory of Theoretical Biophysics, School of Physical Science and Technology, Inner Mongolia University, 235 West Daxue Road, Hohhot 010021, China; Laboratory of Theoretical Biophysics, School of Physical Science and Technology, Inner Mongolia University, 235 West Daxue Road, Hohhot 010021, China; Laboratory of Theoretical Biophysics, School of Physical Science and Technology, Inner Mongolia University, 235 West Daxue Road, Hohhot 010021, China; Laboratory of Theoretical Biophysics, School of Physical Science and Technology, Inner Mongolia University, 235 West Daxue Road, Hohhot 010021, China; Laboratory of Theoretical Biophysics, School of Physical Science and Technology, Inner Mongolia University, 235 West Daxue Road, Hohhot 010021, China; Laboratory of Theoretical Biophysics, School of Physical Science and Technology, Inner Mongolia University, 235 West Daxue Road, Hohhot 010021, China; Laboratory of Theoretical Biophysics, School of Physical Science and Technology, Inner Mongolia University, 235 West Daxue Road, Hohhot 010021, China

## Abstract

The discovery of key epigenetic modifications in cancer is of great significance for the study of disease biomarkers. Through the mining of epigenetic modification data relevant to cancer, some researches on epigenetic modifications are accumulating. In order to make it easier to integrate the effects of key epigenetic modifications on the related cancers, we established CancerMHL (http://www.positionprediction.cn/), which provide key DNA methylation, histone modifications and lncRNAs as well as the effect of these key epigenetic modifications on gene expression in several cancers. To facilitate data retrieval, CancerMHL offers flexible query options and filters, allowing users to access specific key epigenetic modifications according to their own needs. In addition, based on the epigenetic modification data, three online prediction tools had been offered in CancerMHL for users. CancerMHL will be a useful resource platform for further exploring novel and potential biomarkers and therapeutic targets in cancer.

**Database URL**: http://www.positionprediction.cn/

## Introduction

A large number of studies had found that epigenetic modifications play an important role in tumorigenesis (1–3). The abnormal changes of epigenetic modifications may lead to the alterations of genome structure and gene expression and tumorigenesis (4–6). Because epigenetic changes are reversible, the identification of key epigenetic modifications in cancer may be useful for the study of targeted epigenetic therapies (7).

In recent years, with the rapid increase of DNA methylation, histone modifications and lncRNA associated with cancer, some specific resources on DNA methylation, histone modifications, and lncRNAs in diseases have been made. For instance, MethHC 2.0 (8), DiseaseMeth 3.0 (9), MethyCancer (10), MethDB (11), Pancan-meQTL (12) and DDMGD (13) included DNA methylation data obtained from experiments. PubMeth (14) and MeInfoText (15) extracted information about cancer methylation by mining literature. MethMarkerDB focused on some especial genes and methylated regions in cancer (16). HHMD (17) and iHMS (18) were databases on histone modification data obtained from experiments. Lnc2Cancer 3.0 (19), LncRNADisease 3.0 (20), LncTarD 2.0 (21), Lnc2Meth (22), LnCeCell (23), LncSEA 2.0 (24) and lncRNASNP2 (25) integrated the disease-related lncRNA data supported by experiment and computation.

In our previous works, the changes of DNA methylation and histone modification patterns in functional regions were explored by analyzing the DNA methylation and histone modification data in liver cancer, breast cancer, colon cancer, lung cancer and chronic myelogenous leukemia. Based on these changes, the key methylation sites and histone modification regions were discovered in these cancers and the effect of the key epigenetic modification changes on gene expression were deeply discussed.

Based on these databases and our previous studies, it will be useful to construct the platform of the integrated epigenetic modification data. Therefore, we established CancerMHL in which the information related key DNA methylation, histone modifications and lncRNAs in several cancers can be easily obtained. It will provide a useful platform for exploring novel epi-biomarkers and therapeutic targets in the study of these cancers. The current version of CancerMHL collects key methylation sites, histone modifications and their regions as well as lncRNAs related to cancer. Each item includes the detailed annotation information of epigenetic modification, such as regions, sites and their effects on gene expression. Furthermore, based on our previous studies, CancerMHL collected the distribution patterns of differential DNA methylation and differential histone modifications in the various regions of genomes for different cancers. It also provides the several online tools of effective prediction algorithms and risk assessment models.

## Data collection and database content

### Data collection and processing

We extracted key DNA methylation and histone modification data in cancer based on the articles published by our research team (26–34). The main data include: (i) key differential histone modifications and their important regions in cancer, as well as their regulated target genes; (ii) key differential methylation sites in cancer and their regulated target genes; (iii) the effect of key epigenetic modifications on gene expression; (iv) the distribution changes of DNA methylation of up- and down-regulated genes in different functional regions; (v) the important differential expression genes; (vi) the important histone modification regions associated with the different types of gene expressions; (vii) the correlation of different histone modifications in up- and down-regulated genes. For their regulated target genes, we annotated gene types based on TSGene 2.0 (35), ONGene (36), COSMIC (37) and TAG (38) databases. Furthermore, we used the PubMed literature database to verify if these genes have been experimentally confirmed to influence cancer occurrence and development.

Many studies have found that DNA methylation, histone modifications and lncRNAs play a cooperative role in the regulation of gene expression (39–41). To facilitate such research, we searched the PubMed literature database using the keywords ‘cancer,’ ‘gene,’ and ‘lncRNA’. According to the following criteria: (i) lncRNAs related to human diseases and (ii) consistency in target genes regulated by lncRNAs with those regulated by methylation and histone modifications, we retrieved information about lncRNAs and their regulatory mechanisms on target genes. Based on the cooperative regulatory mechanisms of lncRNAs with other regulatory factors on target genes, we categorized lncRNA regulation into the following types: (i) co-regulation with DNA methylation; (ii) co-regulation with histone modifications; (iii) co-regulation with transcription factors; (iv) co-regulation with DNA methylation and histone modifications; and (v) lncRNAs independently regulate target genes.

### Database statistics

The current version of CancerMHL contained key epigenetic modifications from 10 types of cancers, including key DNA methylation sites in different functional regions, the key regions of different histone modifications, and important lncRNAs participated in different co-regulations. The statistical results are shown in Tables 1, 2 and 3, respectively. Furthermore, CancerMHL provided the distribution patterns of DNA methylation and histone modification in different regions by mining epigenetic modification data related to cancer. It also provided two online risk assessment tools and an early diagnosis tool for the convenience of medical researchers.

**Table 1.** The number of key methylation sites in different regions of the genome

Disease	Regions							
Breast cancer	Promoter	Enhancer	Exon	Intron	CGI	N_Shelf	N_Shore	S_Shore
	21	32	6	25	72	5	5	1
Hepatocellular carcinoma	Promoter	5ʹUTR	exon	CGI		N_Shelf	N_Shore	S_Shore
	12	2	7	2		1	8	2
Colon adenocarcinoma	TSS200	TSS1500	body	CGI		N_Shore	S_Shore	5ʹUTR
	2	4	19	5		4	4	1
Pan-cancer	TSS200		TSS1500		Body		CGI	
	1		2		2		1	

Abbreviation: TSS200, 200 bp in the upstream of the transcription start site (TSS). TSS1500, 200–1500 bp in the upstream of the TSS.

**Table 2.** The number of key regions in different histone modifications

Disease	Histone modifications						
Hepatocellular carcinoma	H3K4me1	H3K4me2	H3K4me3	H3K27me3	H3K79me2	H3K9ac	H3K27ac
	2	8	22	5	5	18	28
Breast cancer	H3K36me3		H3K79me2		H3K9ac		H3K27ac
	3		3		6		6
**Disease**	**Histone modifications**	**Disease**	**Histone modifications**		**Disease**		**Histone modifications**
Colorectal cancer	H3K79me3	Lung adenocarcinoma	H3K79me2		Chronic myelogenous Leukemia		H3K36me3
	20			12			6

**Table 3.** The number of important lncRNAs participated in different co-regulations

Disease	Regulation types (numbers)				
Hepatocellular carcinoma	lncRNA (28)	lncRNA-Meth (9)	lncRNA-HM (7)	lncRNA-TF (2)	lncRNA-Meth-HM (1)
Colorectal cancer	lncRNA (17)	lncRNA-Meth (4)	lncRNA-HM (2)	lncRNA-Meth-HM (2)	
Non-small cell lung carcinoma	lncRNA (20)	lncRNA-Meth (1)	lncRNA-HM (3)	lncRNA-TF (5)	
**Disease**	**Regulation types (numbers)**		**Disease**	**Regulation types (numbers)**	
Breast cancer	lncRNA (9)	lncRNA-Meth (1)	Colon adenocarcinoma	lncRNA (2)	
Lung adenocarcinoma	lncRNA (7)	lncRNA-TF (1)	Lung cancer	lncRNA (2)	
Chronic myeloid leukemia	lncRNA (6)	lncRNA-Meth (2)	Lung squamous cell carcinoma	lncRNA (1)	

Abbreviation: Meth, DNA methylation. HM, histone modification.

## Database usage and access

### Web interface

CancerMHL offers a user-friendly web interface that enables user to browse and retrieve the information of epigenetic modifications in cancer. The CancerMHL website comprises three main modules, including (i) ‘Search’ for accessing the information about key epigenetic modifications in cancer, (ii) ‘Conclusion’ for obtaining the distribution patterns of DNA methylation and histone modifications in cancer and (iii) ‘Tools’ for using online tools for risk assessment and the early diagnosis of cancer patients. In addition, on the ‘Help’ page, CancerMHL provides a detailed tutorial for the usage of the database.

### Using the search tool to retrieve the key epigenetic modifications in cancer

**Figure 1.**
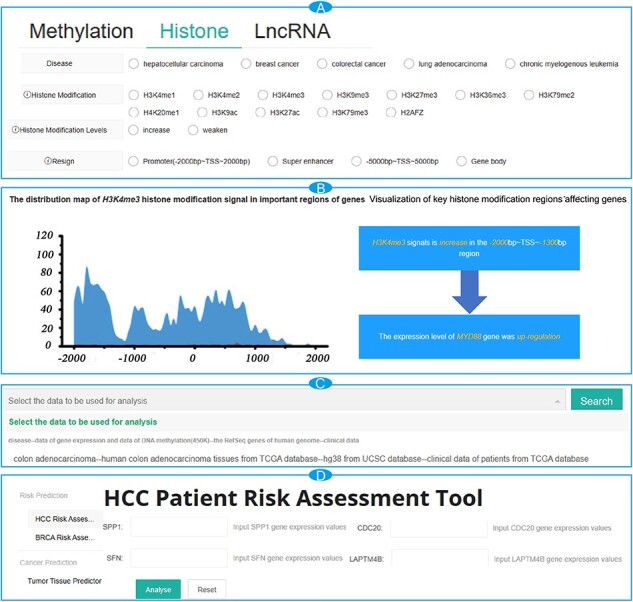
The interface of the browse module for CancerMHL. (A) The interface of the search modules; (B) search result page for MYD88 with detailed information; (C) module interface for analyzing results based on DNA methylation and histone modification data; (D) the interface of the tool modules.

To access interesting key epigenetic modifications, users can query the database as follows. First, on the ‘Search’ page, users can filter data by using the type, disease and the location of the desired key epigenetic modifications (Figure 1A). Once selections are made, click the ‘Search’ button and the query results will be shown as a responsive table, with each item representing information about a key epigenetic modification. There is a ‘Details’ button at the end of each item, which will provide more comprehensive annotation information, including the visual representations of the effects of epigenetic modifications on gene expression, the location details of epigenetic modifications, information about genes and the distribution of signals in important histone modification regions (Figure 1B).

### Retrieving the distribution patterns of DNA methylation and histone modifications in cancer

On the ‘Conclusion’ page, users can explore the distribution patterns of DNA methylation and histone modifications, as well as some relevant important results, by selecting the data used for computational analysis from the dropdown list (Figure 1C).

### Using of risk assessment tools and tumor tissue prediction tools

CancerMHL provides three online tools: a risk assessment tool for liver cancer patients, a risk assessment tool for breast cancer patients and a tumor tissue prediction tool. On the ‘Tools’ page, users select their interested tool from the options on the left side of the page. Following the instructions provided on the page, the users enter patient data into the input boxes (Figure 1D). Subsequently, by clicking the ‘Analyze’ button, users can obtain the patient’s risk grouping or predicted results.

## Conclusions and future extensions

In this study, we established the integrated database of key epigenetic modifications in the several kinds of cancers. This platform provides a reference for further studying the effects of co-regulation with epigenetic modifications such as DNA methylation, histone modifications and lncRNAs on gene expression levels, including the effects of co-regulation with the different epigenetic modifications in same regions or same epigenetic modifications in different regions on gene expression, etc. Moreover, some of the important genes and epigenetic modification regions and sites can be used as potential epigenetic biomarkers. The establishment of this database will have significant guiding value for experimental researchers in exploring novel epigenetic biomarkers and therapeutic targets.

To make CancerMHL more comprehensive and useful, we will further improve and perfect the platform and database, and increase more annotation information and practical analysis tools. We plan to supplement relationship between chromatin accessibility annotation and key epigenetic modifications and the effects of epigenetic modifications on 3D genome structure in future versions.

## Data Availability

All the data can be downloaded from http://www.positionprediction.cn/.
